# The Association of Nursing Home Staffing Levels With Consumer Complaints

**DOI:** 10.1093/geroni/igaa057.2454

**Published:** 2020-12-16

**Authors:** Lindsay Peterson, John Bowblis, Dylan Jester, Kathryn Hyer

**Affiliations:** 1 University of South Florida, Tampa, Florida, United States; 2 Miami University, Oxford, Ohio, United States

## Abstract

Nursing homes (NH) are inspected annually, however, residents and others can file complaints any time. Complaints are critical to NH oversight. Another important quality factor is staffing. Our objective was to examine the association of complaints and staffing levels in a 2017 sample of 14,194 freestanding NHs. We used federal data on NH complaints, quality, staffing, and other characteristics. The outcomes were having received at least one complaint (or not) and numbers of complaints. Using logit and negative binomial regression, controlling for facility and resident characteristics, we found greater registered nurse, nursing assistant, and social services staffing were associated with fewer complaints. Interestingly, licensed practical nurse (LPN) staffing was associated with a higher likelihood of receiving a complaint. Results are consistent with literature on nurse staffing and quality. LPN results raise questions about substituting LPNs for RNs. The social services results show social services staffing may be important for quality.

